# Designing a Synthetic Microbial Community to Enhance Flavor Compound Production in Sesame Flavor-Type *Baijiu* Fermentation

**DOI:** 10.3390/foods15091476

**Published:** 2026-04-23

**Authors:** Xueao Ji, Xiaowei Yu, Yan Xu, Qun Wu

**Affiliations:** 1 School of Life Sciences, Inner Mongolia University, Hohhot 010000, China; 111992070@imu.edu.cn; 2 Lab of Brewing Microbiology and Applied Enzymology, School of Biotechnology, Jiangnan University, Wuxi 214122, China; yuxw@jiangnan.edu.cn (X.Y.); yxu@jiangnan.edu.cn (Y.X.)

**Keywords:** sesame flavor-type *baijiu*, core microbiota, synthetic microbial community, food fermentation

## Abstract

*Fuqu* plays a crucial role in initiating fermentation and flavor compound production during sesame flavor-type *baijiu* fermentation. However, selecting microorganisms for *Fuqu* to enhance flavor compound production remains a challenge. This work designs a synthetic microbial community (SynCom) for *Fuqu* to improve the production of flavor compounds, with a focus on the diversity of flavor compounds and the content of a key flavor compound—sulfur compounds. Through multi-omics analysis, 13 genera (*Aspergillus*, *Bacillus*, *Lactobacillus*, *Leuconostoc*, *Pediococcus*, *Pichia*, *Saccharomyces*, *Trichosporon*, *Weissella*, *Candida*, *Torulaspora*, *Clavispora*, and *Wickerhamomyces*) were identified as core microbiota involved in the production of those flavor compounds, and these core microbiota were used to construct a SynCom to enhance flavor compound production in *baijiu* fermentation. The resulting SynCom exhibited the highest flavor compound diversity (0.64) and 3-(methylthio)-1-propanal content (618.14 μg/kg) in simulative fermentation. In large-scale production, *Fuqu* made with the SynCom achieved greater flavor compound diversity (0.56) and a higher concentration of 3-(methylthio)-1-propanal (590 μg/kg) compared to commercial *Fuqu* (0.40 and 324 μg/kg, respectively) (*p* < 0.05). The results demonstrated that the SynCom developed for *Fuqu* effectively enhances the production of flavor compounds. This work provides a strategy for constructing SynCom to improve the formation of flavor compounds in *baijiu* fermentation.

## 1. Introduction

Sesame flavor-type *baijiu*, produced through solid-state fermentation by complex microbiota, is famous for its unique roasted aroma [1]. *Fuqu*, composed of a limited number of pure-culture microorganisms, serves as a crucial starter culture that supplies microbiota for fermentation [2]. Microbial metabolism during *baijiu* fermentation is the main source of flavor compounds, which are closely associated with *baijiu* quality [3]. Therefore, the selection of microorganisms in *Fuqu* is critical for improving the quality of sesame flavor-type *baijiu*.

*Fuqu* comprises yeast, bacteria, and mold, with the yeast primarily including *Saccharomyces cerevisiae* (*S. cerevisiae*), and *Saccharomycopsis fibuligera* (*S. fibuligera*) [4]. These yeasts are responsible for the metabolism of ethanol and flavor compounds. The bacteria mainly consist of *Bacillus* species, such as *Bacillus licheniformis* (*B. licheniformis*) and *Bacillus amyloliquefaciens* (*B. amyloliquefaciens*), providing proteases and flavor precursors during fermentation [5]. The mold is dominated by *Aspergillus* and *Rhizopus* species, whose secretion of acidic protease hydrolyzes proteins and thus generates precursors of flavor compounds [2]. However, the selection of microorganisms in *Fuqu* is largely random, leading to variability in the quality of sesame-flavor-type *baijiu*, as reflected by insufficient levels of flavor compounds.

In addition, the non-standardized selection of microorganisms for *Fuqu* also results in a lack of sesame aroma. Sulfur compounds are widely present in fermented foods characterized by strong sensory impacts. As a crucial class of odor-active compounds, sulfur compounds directly shape the flavor profiles of fermented foods and beverages, contributing to characteristic notes, including pickled, fruity [6], meaty [7], and sesame aromas [8]. Previous studies have identified sulfur compounds as key contributors to sesame aroma, such as 3-(methylthio)-1-propanal, 2-furfurylthiol, and dimethyl trisulfide [9,10,11]. Moreover, sulfur compounds usually exist at trace levels (ng/L or ng/kg) and possess low detection thresholds, making their qualitative and quantitative analysis extremely challenging. For this reason, most targeted analytical methods are based on derivatization [12]. Microbial metabolism is an important source of sulfur compounds in *baijiu* fermentation [13]. Previous research on sulfur compounds in sesame flavor-type *baijiu* has focused on their qualitative and quantitative analysis [14]. However, the selection of microorganisms for *Fuqu* to enhance sulfur production remains poorly understood. Among sulfur compounds, 3-(methylthio)-1-propanal is a key precursor of other sulfur compounds, such as 3-(methylthio)-1-propanol, methanethiol, dimethyl disulfide, and dimethyl trisulfide [15].

The challenge of selecting appropriate microorganisms for *Fuqu* lies in the rational construction of a synthetic microbial community (SynCom) for sesame flavor-type *baijiu* fermentation, yet few studies have explored this development. The bottom-up approach to SynCom construction has gained considerable attention in food fermentation [16]. It typically involves the use of sequencing technologies to uncover the composition or functional traits of complex microbial communities, the isolation of pure cultures via culturomics, and the subsequent assembly of selected microorganisms according to defined functional objectives [17]. Studies on SynCom for fermented foods can be broadly categorized into two types. The first aims to enhance the production of specific target compounds. For example, a seven-strain SynCom was constructed to increase the content of pyrazines [18]. The second focuses on mimicking the flavor profile of native fermentation systems as illustrated by SynCom construction in baijiu, vinegar, and vegetable fermentations [19,20,21]. However, moving beyond mimicry to construct SynComs that actively enhance overall flavor compound production remains a major challenge.

In this study, we aimed to construct a SynCom for *Fuqu* to enhance flavor compound production, including flavor compound diversity and a key flavor compound—a sulfur compound, during sesame flavor-type *baijiu* fermentation. Through amplicon sequencing, metatranscriptomic, and metagenomic analyses, we identified core microbiota and constructed a SynCom accordingly. We hypothesize that the constructed SynCom enhances the diversity of flavor compounds and the production of sulfur compounds in both simulated fermentation and large-scale production. This work provides a strategy for designing SynComs to enhance flavor compound formation in complex fermentation systems.

## 2. Materials and Methods

### 2.1. Sample Collection

Fifty-eight samples collected from the four different sesame flavor-type *baijiu* fermentations (SF, JY, JQA, JQB) were obtained from our previous study [22]. For each fermentation, triplicate independent fermentation batches were sampled. Samples were collected from the upper layer (0.5 m deep) at 0, 5, 10, 20, and 30 days. The current work is a follow-up study aimed at identifying the core microbiota based on taxonomic composition across four fermentations.

Among the four *baijiu* fermentations, SF exhibited the highest diversity of flavor compounds [22] and thus was further selected to identify transcriptionally active microbiota and core microbiota associated with flavor compound production. The SF fermentation samples used for this identification were obtained from a previous study [23]. Briefly, six samples were collected at early (8 d) and late (15 d) stages of SF fermentation, with three samples taken from distinct fermentation pits at each time point.

Fermentation samples (5 d), characterized by high production of 3-(methylthio)-1-propanal, were used to investigate the core microbiota involved in sulfur compound production. The simulated fermentation was conducted at the JY sesame flavor-type *baijiu* distillery. These samples were derived from our recently published study [13].

### 2.2. Total DNA Extraction, Amplification, and Sequencing

The total DNA of fermented grains, extracted using a soil DNA kit (Omega Bio-Tek, Norcross, GA, USA), was used for high-throughput sequencing to reveal microbial composition. Bacterial 16S rRNA gene (V3–V4 region) and fungal 18S rRNA gene (ITS1/ITS2 region) were amplified using primers 338F/806R and ITS2/ITS3, respectively [24,25]. Amplification products were sequenced on an Illumina Miseq Sequencer (Illumina, San Diego, CA, USA).

All raw sequences were processed using QIIME (V. 1.9.1) [22]. Briefly, sequences were filtered if they contained more than two ambiguous bases, had a quality score below 30, included more than 10 homopolymers, showed primer mismatches, or were shorter than 200 bp. Chimeric sequences were removed with UCHIME [26]. High-quality sequences were clustered into operational taxonomic units (OTUs) based on a 97% identity threshold [27]. The taxonomic annotations were assigned to the EzBioCloud and UNITE databases.

### 2.3. Metagenomic Sequencing

The DNA quality was determined using a NanoDrop ND-2000 spectrophotometer (Thermo Scientific, Waltham, MA, USA). The DNA library was prepared using the TruSeqTM DNA Sample Prep Kit and sequenced on the Illumina HiSeq 4000 platform (Illumina, San Diego, CA, USA). All operations were carried out according to the manufacturer’s instructions. After quality filtering of raw reads using SeqPrep and Sickle (Q-score ≥ 30) [28], the resulting sequences were assembled with IDBA-UD v1.1.1 [29], and sequences shorter than 150 bp were subsequently removed. CD-HIT was used to cluster the filtered gene sequences based on 95% sequence similarity [30].

### 2.4. RNA Extraction and Metatranscriptomic Sequencing

The total RNA of fermented grains was extracted using a soil RNA kit (Omega Bio-Tek, Norcross, GA, USA). RNase-free DNase (Qiagen, Hilden, Germany) was used to remove residual DNA. The total RNA quality and concentration were assessed using a NanoDrop 2000 spectrophotometer (Thermo Scientific, Waltham, MA, USA). RNA libraries were prepared using the TruSeq RNA Sample Preparation Kit (Illumina, San Diego, CA, USA). The RNA library was sequenced on the Illumina HiSeq 4000 platform (Illumina, San Diego, CA, USA).

Raw sequences were filtered using SeqPrep and Sickle. Sequences shorter than 50 bp, with an average quality score below 20, or containing ambiguous bases (N) were removed [20]. TopHat2 software (2.1.1) was used to remove contaminant sequences that showed high similarity to host DNA sequences [31]. SortMeRNA software (v4.3.3) was employed to remove rRNA reads, using the SILVA SSU and SILVA LSU databases as references [32]. High-quality reads were assembled using the Trinity software (v2.11.0), and assembled sequences longer than 300 bp were retained for further analysis. Gene prediction of assembled sequences was conducted using Trans GeneScan (v1.3.0). A nonredundant gene set was then clustered with CD-HIT, applying a threshold of >95% sequence similarity and >90% coverage [30]. Gene expression levels were quantified as FPKM using RSEM software (v1.3.3) [33].

### 2.5. SynCom Design

The identification of SynCom members was guided by taxonomic composition and transcriptional activity, as well as core microbiota involved in flavor compound diversity and sulfur compound production. The SynCom comprised 13 microbial genera, including *Pichia*, *Bacillus*, *Aspergillus*, *Lactobacillus*, *Saccharomyces*, *Wickerhamomyces*, *Candida*, *Weissella*, *Pediococcus*, *Leuconostoc*, *Trichosporon*, *Clavispora*, and *Torulaspora*.

*Pichia*, *Bacillus*, *Aspergillus*, *Saccharomyces*, and *Wickerhamomyces* are microorganisms commonly used in the preparation of *Fuqu* [5,34], and are known to be key contributors to starch degradation, ethanol production, and flavor compound formation [35]. Therefore, these 5 genera were selected to construct SC1, aiming to mimic the natural species composition of *Fuqu*. *Lactobacillus*, *Leuconostoc*, *Weissella*, and *Pediococcu* are the most common genera of lactic acid bacteria, which produce a large number of flavor compounds or precursors, such as lactic acid, acetic acid, and pyruvate [36]. SC2 was constructed by adding four lactic acid bacteria genera to the five microorganisms present in SC1, with the aim of enhancing the formation of flavor compounds. SC3 encompassed all 13 core microbial genera and included four additional yeasts compared to SC2, allowing us to assess the impact of increased yeast complexity on the production of flavor compounds.

High-abundance microorganisms from the 13 genera were isolated. These strains included *Pichia kudriavzevii* (*P. kudriavzevii*), *B. amyloliquefaciens*, *Aspergillus oryzae* (*A. oryzae*), *S. cerevisiae*, *Wickerhamomyces anomalus* (*W. anomalus*), *Lactobacillus acetotolerans* (*L. acetotolerans*), *Weissella paramesenteroides* (*W. paramesenteroides*), *Pediococcus acidilactici* (*P. acidilactici*), *Leuconostoc pseudomesenteroides* (*L. pseudomesenteroides*), *Candida vini* (*C. vini*), *Trichosporon asahii* (*T. asahii*), *Clavispora lusitaniae* (*C. lusitaniae*), and *Torulaspora delbrueckii* (*T. delbrueckii*).

### 2.6. Isolation of Microorganisms

Fermented grains collected on days 5 and 10 of sesame flavor-type *baijiu* fermentation were used to isolate microorganisms. Briefly, 10 g of fermented grains were mixed with 40 mL of sterile water and shaken for 30 min (200 rpm, 30 °C). The supernatant was then serially diluted and plated onto Luria–Bertani (LB), de Man, Rogosa, and Sharpe (MRS), and potato dextrose agar (PDA) media. *Bacillus* sp. were isolated on LB medium with incubation at 37 °C for 48 h. Lactic acid bacteria were cultured on MRS medium at 37 °C for 48–72 h. Fungi were grown on PDA medium at 30 °C for 24–120 h. Bacterial isolates were identified using 16S rRNA gene sequencing with universal primers 27F (5′-AGAGTTTGATCMTGGCTCAG-3′) and 1492R (5′-CGGTTACCTTGTTACGACTT-3′) [37], while fungal isolates were characterized by ITS region sequencing using primers ITS1 (5′-TCCGTAGGTGAACCTGCGG-3′) and ITS4 (5′-TCCTCCGCTTATTGATATGC-3′) [38]. The obtained sequences were then compared with the National Center for Biotechnology Information (NCBI) database for microbial identification. The results of strain identification are shown in Table S1. The isolated strains were prepared as glycerol stocks (25% final glycerol concentration) and stored at −80 °C for long-term preservation.

### 2.7. Simulated Fermentation

All strains were incubated in the sorghum extract medium. Yeast was incubated at 30 °C for 24 h with shaking at 150 rpm. Bacteria (except for *L. acetotolerans*) were incubated at 37 °C for 14 h with shaking at 150 rpm. *L. acetotolerans* was cultured anaerobically at 37 °C for 3 d. *A. oryzae* was incubated at 30 °C for 5 d. Each strain in the SynComs was mixed at an equal concentration of 1 × 10^5^ CFU/mL.

The simulated fermentation was performed at a sesame flavor-type *baijiu* distillery located in Suqian City, Jiangsu Province, China. Fermented grains from the last batch were blended with fresh grains at a mass ratio of 4.5:1 [13]. A total of 500 g of mixed materials was placed into fermentation bags. After steaming and cooling, the SynCom was inoculated into the materials. The fermentation bags were then embedded in the upper layer of the pit (0.5 m deep) to simulate actual *baijiu* fermentation for 30 d. Following the completion of fermentation, all samples were immediately frozen in liquid nitrogen and stored at −80 °C.

### 2.8. Large-Scale Production Fermentation

Based on the results of simulated solid-state fermentation, the optimal SynCom was selected for large-scale production fermentation. This fermentation was carried out at a sesame flavor-type *baijiu* distillery located in Suqian City, Jiangsu Province, China. The SynCom was used to prepare *Fuqu* following the standard procedures of the production workshop at the sesame flavor-type *baijiu* distillery.

The SynCom members were categorized into three groups: bacteria, yeast, and mold, which were then separately prepared into *Fuqu*. For bacteria, a colony from a slant was inoculated into LB medium (300 mL) and cultured at 37 °C, 150 rpm for 18 h, while *L. acetotolerans* was grown in MRS medium statically at 37 °C for 72 h. All bacterial strains were then co-cultured in a 40 L sterile fermenter at 37 °C with stirring for 18 h, followed by scale-up in a 400 L fermenter for another 18 h. The resulting suspension was sprayed onto steamed wheat bran (5% inoculation), mixed, and incubated statically at 35–40 °C for 24–36 h. Yeast from slant cultures was transferred to 300 mL PDA medium and shaken at 30 °C, 150 rpm for 24 h. The cultures were combined in a 40 L sterile fermenter for 24 h at 30 °C with stirring, then scaled up in a 400 L fermenter for another 24 h. The final suspension was applied to steamed wheat bran (5% inoculation), evenly mixed, and statically incubated at 30–35 °C for 24–36 h. Mold from slants was sprayed onto steamed wheat bran at 0.3% inoculation and incubated statically at 30–35 °C for 3–5 days.

The commercial *Fuqu* used in routine production at the same distillery served as the control. The SynCom and control fermentations were each set up in three batches. Six samples were collected from the upper-layer pit (0.5 m deep) on day 30 of fermentation. All samples were stored at −80 °C for subsequent analysis.

### 2.9. Detection of Ethanol and Flavor Compounds

Five grams of fermented grains were mixed with 20 mL of double-distilled water and subjected to ultrasonic treatment for 30 min at 0 °C. The mixture was then centrifuged at 8000× *g* for 10 min at 4 °C, and the supernatant was collected [19]. One milliliter of supernatant was filtered through a 0.2 μm pore-size filter and then used to measure ethanol content by high-performance liquid chromatography (HPLC; Agilent 1200, Milford, MA, USA). Separation was performed on an Aminex HPX–87H column (Bio-Rad, Hercules, CA, USA) with a refractive index detector (RID; Waters 2414, Milford, MA, USA). The column temperature was maintained at 60 °C, with an injection volume of 10 μL. Elution was carried out using 3 mM H_2_SO_4_ as the mobile phase at a flow rate of 0.6 mL/min for 30 min [13].

Subsequently, 8 mL of supernatant was mixed with 20 µL of an internal standard solution (menthol, 108 µg/mL) and 3 g of NaCl, followed by analysis using the headspace solid-phase microextraction gas chromatography-mass spectrometry (HS-SPME-GC–MS) method [39]. Flavor compounds were analyzed using an Agilent 6890 GC system equipped with an Agilent 5975 MS detector (Agilent Technologies, Santa Clara, CA, USA). Separation was performed on a DB-Wax column (30 m × 0.25 mm i.d., 0.25 μm film thickness; J&W Scientific, Folsom, CA, USA). A PDMS-coated solid-phase microextraction fiber (J&W Scientific, Folsom, CA, USA) was used for extraction. Flavor compounds were detected in full-scan mode with a mass scan range of 35–450 amu. The oven temperature program was as follows: initially held at 50 °C for 2 min, ramped to 230 °C at a rate of 6 °C/min, and maintained at 230 °C for an additional 20 min. Helium was used as the carrier gas (2 mL/min) [40]. Flavor compounds were identified based on comparing the spectral matching factor (>75%) between mass spectrometric information of each chromatographic peak and the National Institute of Standards and Technology (NIST) mass spectra library and the difference between RIcal and RIlit (<30 units) [34]. Flavor compound diversity was calculated by normalizing the contents of flavor compounds across all fermented samples to a 0–1 range, followed by averaging the normalized values for each sample [22].

### 2.10. Detection of 3-(Methylthio)-1-propanal

3-(Methylthio)-1-propanal is a critical precursor of volatile sulfur compounds in baijiu fermentation and contributes markedly to the roasted note of sesame-flavor baijiu [10]. Detection of 3-(Methylthio)-1-propanal was as follows: 10 g of fermented grains were combined with 20 mL of distilled water, and the supernatant was collected. Eight milliliters of supernatant were mixed with 20 µL of an internal standard solution (menthol, 10 µg/mL) and 3 g of NaCl [13]. The detection of 3-(Methylthio)-1-propanal was carried out using the headspace solid-phase microextraction gas chromatography-pulsed flame photometric (HS-SPME-GCPFPD) method [8]. The detection instrument was an Agilent 7890 A gas chromatography system equipped with a pulsed flame photometric detector (OI Analytical model 5380, OI Analytical Co., College Station, TX, USA). The separation column was DB-FFAP (30 m by 0.32 mm inside diameter [i.d.], 1-μm film thickness; J&W Scientific, Folsom, CA, USA). The instrument conditions were applied as previously described [13]. The 3-(methylthio)-1-propanal standard was used to prepare a calibration curve, and the content of this compound was quantified by substituting its peak area into the curve.

## 3. Results

### 3.1. Microbial Composition During Sesame Flavor-Type Baijiu Fermentations

**Figure 1:**
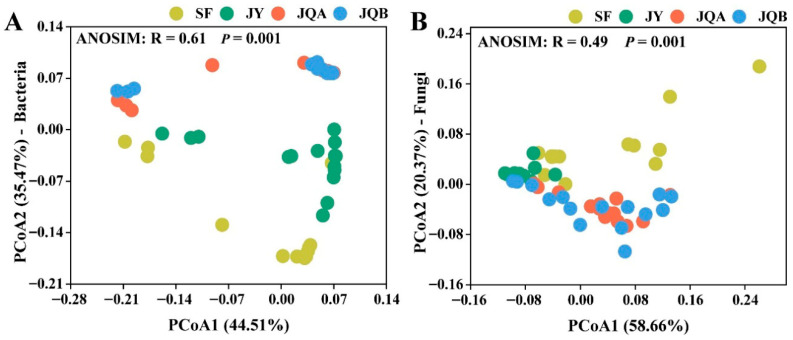
PCoA revealed phylogenetic clustering of the dominant (**A**) bacterial and (**B**) fungal genera as tested by ANOSIM. SF, JY, JQA, and JQB represent different sesame flavor-type *baijiu* fermentations, respectively.

Given that sesame flavor-type *baijiu* is produced across multiple distilleries and exhibits differences in quality, 58 samples were collected from four fermentations of three famous *baijiu* distilleries. Amplicon sequencing was used to characterize the microbial community composition shared across the four fermentations. The bacterial and fungal OTUs with relative abundance greater than 1% were identified as dominant microorganisms. These OTUs were assigned to 11 bacterial and 16 fungal genera, as reported in our previous work [22]. Dominant microbial genera were chosen for principal coordinates analysis (PCoA) to reveal variations in microbial composition. The microbial composition of four fermentations separated along the first principal coordinates. In bacterial and fungal communities, the first two principal coordinates accounted for 79.98% and 79.03% of the total variance, respectively. The bacterial communities exhibited greater variability between groups compared to the fungal communities, as reflected by the dispersion of bacterial samples. ANOSIM analysis revealed significant differences in bacterial composition among the four fermentations (Bacteria: R = 0.61, *p* = 0.001). (Figure 1A). For the fungal communities, although the samples clustered more closely, distinct group-level differences were still observed in the ordination space, as supported by the statistical test results (ANOSIM: R = 0.49, *p* = 0.001) (Figure 1B).

### 3.2. Identification of Core Microbiota Based on Taxonomic Composition and Transcriptional Activity During Sesame Flavor-Type Baijiu Fermentation

**Figure 2:**
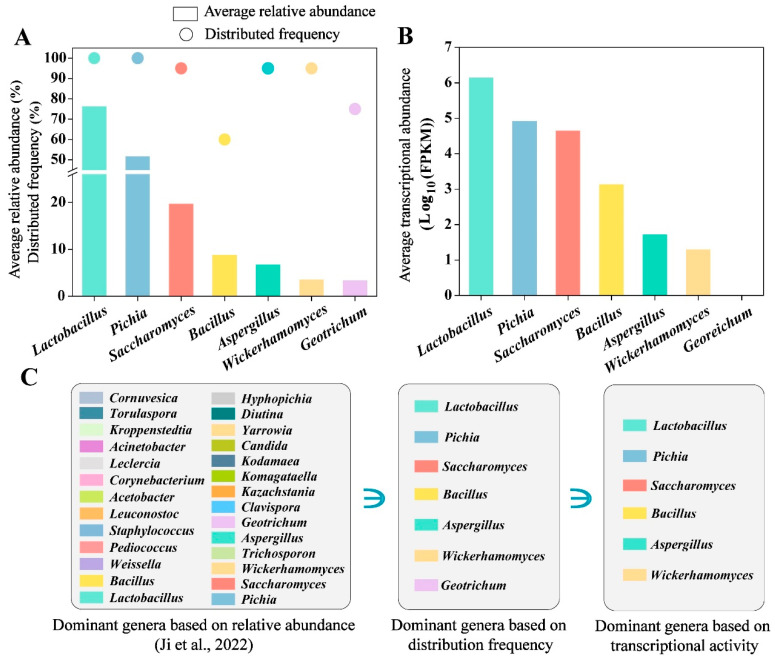
Identification of core microbiota in the fermentation of sesame flavor-type *baijiu*. (**A**) Identification of dominant abundance and high-frequency distributed genera based on four *baijiu* fermentations. (**B**) Identification of transcriptional activity genera based on SF fermentation. (**C**) Identification of core microbiota based on relative abundance (>1%) [22], distribution frequency (>50%), and transcriptional activity (>8 FPKM).

The distribution frequency is a common method for identifying core microbiota in fermentation systems [19]. Therefore, we further calculated the distribution frequency of these dominant OTUs across the four *baijiu* fermentations. A total of seven microbial genera exhibited a distribution frequency exceeding 50% and an average relative abundance above 1%, including *Lactobacillus*, *Pichia*, *Saccharomyces*, *Bacillus*, *Aspergillus*, *Wickerhamomyces*, and *Geotrichum* (Figure 2A).

Focusing on SF fermentation due to its highest diversity of flavor compounds [22], we used metatranscriptomics to explore the active microorganisms in *baijiu* fermentation. Among the seven dominant genera identified based on distribution frequency, six exhibited transcriptional activity, with each showing an average transcriptional abundance greater than 8 FPKM. The average FPKM values were 1.38 × 10^6^ for *Lactobacillus*, 8.28 × 10^4^ for *Pichia*, 4.41 × 10^4^ for *Saccharomyces*, 1.35 × 10^3^ for *Bacillus*, 51.80 for *Aspergillus*, and 19.81 for *Wickerhamomyces* (Figure 2B). By integrating data from four *baijiu* fermentations using amplicon and metatranscriptomic sequencing, these six microbial genera were identified as core microbiota based on relative abundance (>1%) as reported in our previous study [22], distribution frequency (>50%), and transcriptional activity (>8 FPKM) (Figure 2C).

### 3.3. Identification of Core Microbiota Associated with Flavor Compound Diversity and Sulfur Production

**Figure 3:**
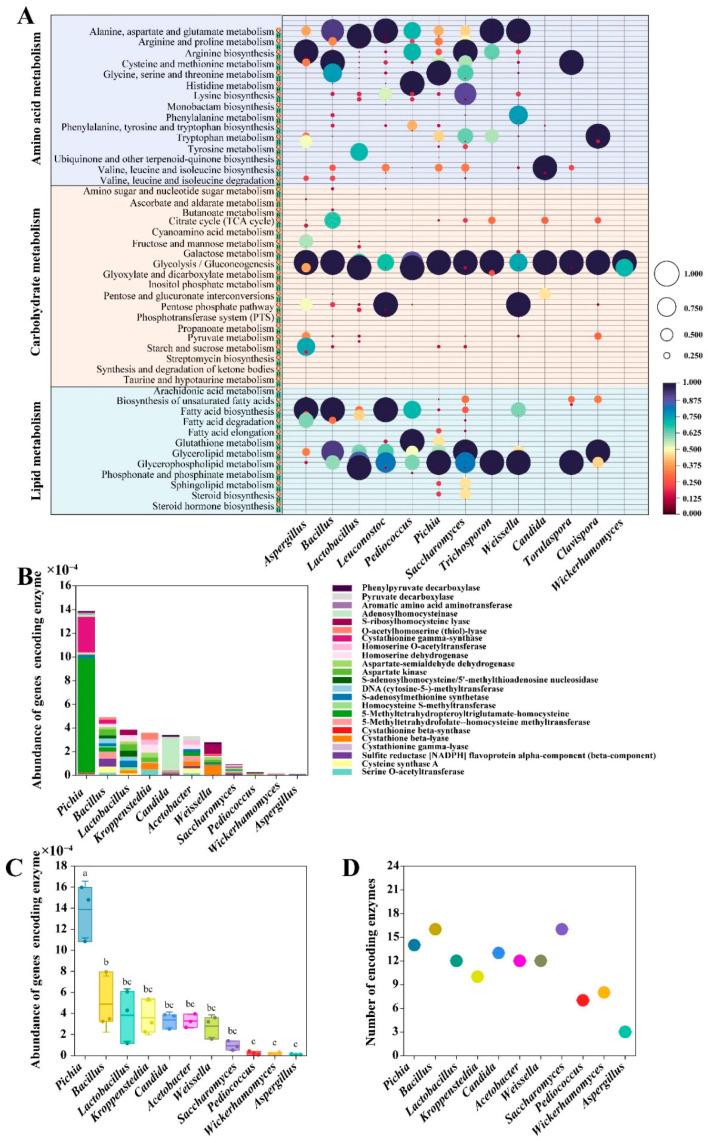
Metatranscriptomic and metagenomic analyses identified core microbiota involved in flavor compound diversity and sulfur compound production during sesame flavor-type *baijiu* fermentation. (**A**) Transcript-active genera and their functional distribution in the amino acid metabolism, carbohydrate metabolism, and lipid metabolism during sesame flavor-type *baijiu* fermentation. Microbial transcriptional abundances in these metabolic pathways were normalized to the range [0, 1]. ‘Q’ and ‘H’ represent early and late fermentation stages, respectively. Color intensity indicates the normalized transcriptional abundance. (**B**) Distribution of genes encoding sulfur metabolic enzymes within core microbiota. (**C**) The total abundance of genes encoding enzymes involved in sulfur production in core microbiota. Significant differences (*p* < 0.05) were determined by Fisher’s LSD test and are indicated by different letters. (**D**) The number of catalytic enzymes associated with sulfur production in core microbiota.

Amino acid, carbohydrate, and lipid metabolism by the microbial community during *baijiu* fermentation are the main pathways for the production of flavor compounds. This work used metatranscriptomics to identify core microbiota associated with these three metabolic processes and to characterize them as contributors to the production of flavor compound diversity. During sesame flavor-type *baijiu* fermentation, 13 microbial genera, including *Aspergillus*, *Bacillus*, *Lactobacillus*, *Leuconostoc*, *Pediococcus*, *Pichia*, *Saccharomyces*, *Trichosporon*, *Weissella*, *Candida*, *Torulaspora*, *Clavispora*, and *Wickerhamomyces*, exhibited transcriptional activity (FPKM > 8) in the metabolic pathways of amino acids, carbohydrates, and lipids. Hence, these 13 microbial genera were core microbiota involved in the production of flavor compound diversity (Figure 3A).

By focusing on 3-(methylthio)-1-propanal, a crucial precursor of other sulfur compounds, our recent work employed metagenomics to reveal 11 core microbiota associated with sulfur production as reported in our recent work [13]. In this work, we further analyzed the abundance of genes encoding enzymes associated with sulfur production in these 11 microorganisms. The distribution of genes encoding sulfur metabolic enzymes within core microbiota is shown in Figure 3B. The total abundance of genes was the highest in *Pichia* (1.39 × 10^−3^), followed by *Bacillus* (4.89 × 10^−4^), *Lactobacillus* (3.84 × 10^−4^), *Kroppenstedtia* (3.58 × 10^−4^), *Candida* (3.39 × 10^−4^), *Acetobacter* (3.29 × 10^−4^), *Weissella* (2.79 × 10^−4^), *Saccharomyces* (9.20 × 10^−5^), *Pediococcus* (2.29 × 10^−5^), *Wickerhamomyces* (1.77 × 10^−5^), and *Aspergillus* (1.13 × 10^−5^) (Figure 3C). The number of catalytic enzymes associated with sulfur production ranged from 3 to 16 across the microorganisms. *Bacillus* and *Saccharomyces* had the highest counts (16 each), whereas *Aspergillus* had the lowest, with only 3 catalytic enzymes (Figure 3D).

### 3.4. Construction of SynComs and Assessment of Their Impact on Flavor Compound Diversity and Sulfur Production in Simulated Fermentation

**Figure 4:**
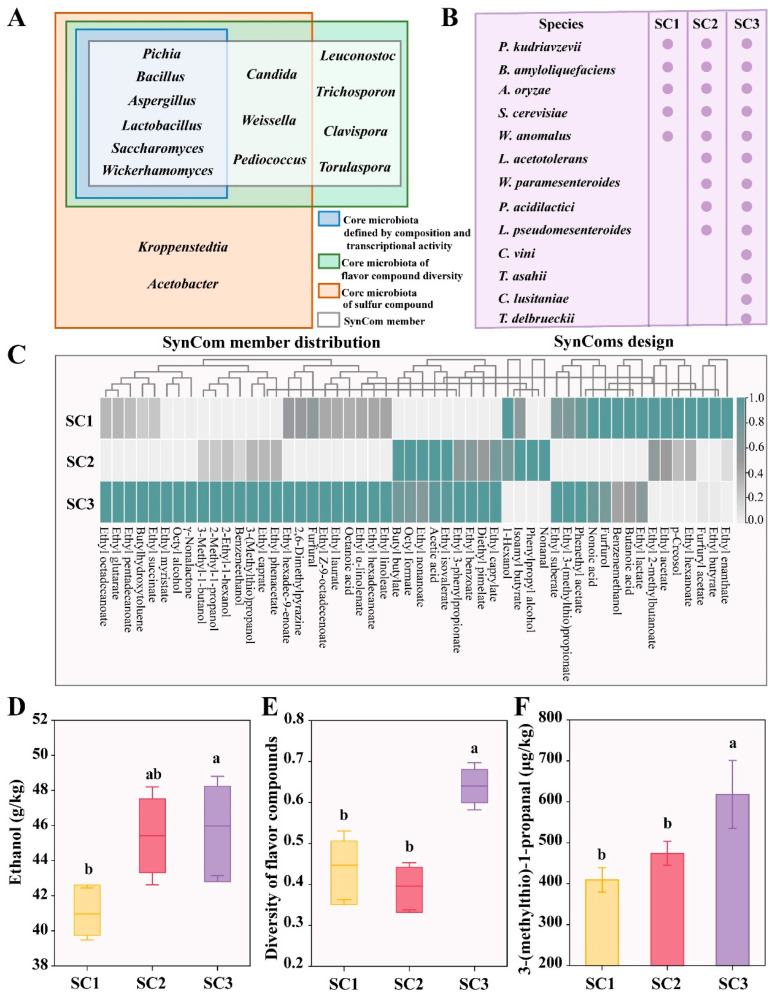
Construction of SynComs and evaluation of their performance in simulated fermentation. (**A**) The distribution of SynCom members. (**B**) Three SynComs (SC1–3) were designed for simulated fermentation. The best-performing SynCom was used for large-scale production. (**C**) Composition of flavor compounds, (**D**) ethanol concentration, (**E**) diversity of flavor compounds, and (**F**) 3-(methylthio)-propanal concentration from SC1, SC2, and SC3 in simulated fermentation. The data for flavor compounds were Z-score transformed. Different letters indicate significant differences (*p* < 0.05) as determined by Fisher’s LSD test.

In this work, we proposed a strategy for constructing a SynCom for sesame flavor-type *baijiu* fermentation. The selection of SynCom members was guided by core microbiota identified through taxonomic composition and transcriptional activity, as well as core microbiota involved in flavor compound diversity and sulfur compound production (Figure 4A). Although *Aspergillus* had a low sulfur production capacity, it was identified as core microbiota based on its high relative abundance and its association with flavor compound production. *Aspergillus* acts as both a saccharifying agent and flavor compound producer in *baijiu* fermentation [3]. Therefore, *Aspergillus* was included in the SynCom. In contrast, *Kroppenstedtia* and *Acetobacter* were high-sulfur-yielding microorganisms, but they were not identified as core microbiota based on relative abundance or flavor compound association and were thus excluded from the SynCom. Therefore, the SynCom consisted of 13 microbial genera: *Pichia*, *Bacillus*, *Aspergillus*, *Lactobacillus*, *Saccharomyces*, *Wickerhamomyces*, *Candida*, *Weissella*, *Pediococcus*, *Leuconostoc*, *Trichosporon*, *Clavispora*, and *Torulaspora*. High-abundance microorganisms from the 13 genera were isolated. These strains included *P. kudriavzevii*, *B. amyloliquefaciens*, *A. oryzae*, *S. cerevisiae*, *W. anomalus*, *L. acetotolerans*, *W. paramesenteroides*, *P. acidilactici*, *L. pseudomesenteroides*, *C. vini*, *T. asahii*, *C. lusitaniae*, and *T. delbrueckii* (Figure 4B).

*P. kudriavzevii* plays a key role in fermentation by contributing to acids and esters [19]. *B. amyloliquefaciens* promotes tetramethylpyrazine formation in sesame flavor-type *baijiu* fermentation [41]. *A. oryzae* secretes glucoamylase and α-amylase, facilitating the production of esters, alcohols, and acids [35]. *S. cerevisiae* is essential for ethanol production in *baijiu* fermentation [19]. *W. anomalus* generates several important flavor compounds, such as ethyl acetate, isobutanol, and furfural [42]. *L. acetotolerans* functions as a core functional microorganism across multiple *baijiu* fermentations [19,23]. *W. paramesenteroides*, *P. acidilactici*, and *L. pseudomesenteroides* are important lactic acid bacteria that produce various flavor compounds and precursors, such as phenylethyl alcohol, isovaleric acid, acetoin, and acetaldehyde [43,44,45]. Additionally, *C. vini* enhances fatty acid production [13], while *T. asahii* exhibits proteolytic activity [46]. Furthermore, *C. lusitaniae* and *T. delbrueckii* contribute significantly to flavor compound production [47]. Therefore, we chose these 13 species for the SynCom experiment.

SynComs (SC1, SC2, SC3) were used for simulated fermentation (Figure 4B). The composition and content of flavor compounds at the end of simulated fermentation in SC1, SC2, and SC3 were determined, and a total of 52 compounds were identified (Figure 4C). Ethanol, a key indicator of *baijiu* fermentation, was measured at the end of the process. It was significantly different among the three SynComs (*p* < 0.05). The ethanol concentration ranged from 40.96 to 45.97 g/kg, with SC1 (40.96 g/kg), SC2 (45.42 g/kg), and SC3 (45.97 g/kg) (Figure 4D). The diversity of flavor compounds in SC1, SC2, and SC3 was 0.45, 0.40, and 0.64, respectively. The diversity in SC3 was significantly higher than that in SC1 and SC2 (*p* < 0.05) (Figure 4E).

We further quantified the 3-(methylthio)-1-propanal content at the end of the three SynCom fermentations. The concentration was 408.98 μg/kg, 473.85 μg/kg, and 618.14 μg/kg in SC1, SC2, and SC3, respectively. Among them, SC3 exhibited a significantly higher level of 3-(methylthio)-1-propanal compared to SC1 and SC2 (*p* < 0.05) (Figure 4F). The above results suggest that the SC3 exhibited the greatest metabolic capacity for ethanol content, flavor compound diversity, and sulfur production.

### 3.5. Effect of SynCom on Flavor Compound Diversity and Sulfur Production in Large-Scale Production

**Figure 5:**
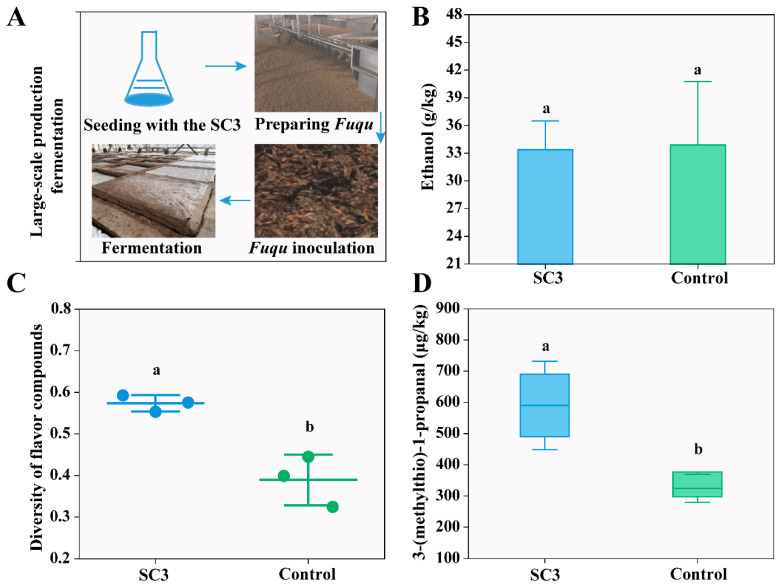
The application of SynCom in large-scale production fermentation. (**A**) The best-performing SynCom (SC3) was selected to be made into *Fuqu* for large-scale production in a famous *baijiu* distillery. (**B**) Ethanol concentration, (**C**) diversity of flavor compounds, and (**D**) 3-(methylthio)-propanal concentration from SC3 and the control group in large-scale production. Fermentation carried out by commercial *Fuqu* served as the control group. Different letters indicate significant differences (*p* < 0.05) as determined by Fisher’s LSD test.

SC3 was subsequently selected to be made into *Fuqu* for large-scale production of *baijiu* (Figure 5A). The commercial *Fuqu* served as the control group. The ethanol content showed no significant difference between SC3 (33.38 ± 7.63% g/kg) and the control group (33.90 ± 8.14% g/kg, *p* > 0.05) (Figure 5B). A total of 102 chromatographic peaks were detected under the full-scan mode of GC–MS, among which 41 flavor compounds were identified. The composition and content of flavor compounds measured at the end of SC3 and control fermentations are shown in Figure S1. The flavor compound diversity and 3-(methylthio)-1-propanal content were analyzed to further evaluate the potential of SC3 to enhance fermentation performance. The diversity of flavor compounds in SC3 was 0.56 ± 3.28%, which was significantly higher than that in the control group (0.40 ± 4.63%; *p* < 0.05) (Figure 5C). Compared to the control group (324 ± 13.94% μg/kg), the 3-(methylthio)-1-propanal content in the SC3 (590 ± 15.07% μg/kg) was significantly increased (*p* < 0.05) (Figure 5D). The results indicate that SC3 not only enhanced flavor compound diversity and sulfur production but also maintained the stability of ethanol production. In addition, SC3 successfully transitioned from simulated fermentation to large-scale production.

## 4. Discussion

The core microbiota in food fermentation are crucial in determining the quality of fermented products [48]. In this work, we identified the core microbiota in sesame flavor-type *baijiu* fermentation based on their taxonomic composition, transcriptional activity, and key roles in flavor compound diversity and sulfur production (Figure 2 and Figure 3). We found that the core microbiota (*Lactobacillus*, *Pichia*, *Saccharomyces*, *Bacillus*, *Aspergillus,* and *Wickerhamomyces*) identified by taxonomic composition and transcriptional activity represented only a subset of that associated with flavor compound diversity and sulfur production, which included *Aspergillus*, *Bacillus*, *Lactobacillus*, *Leuconostoc*, *Pediococcus*, *Pichia*, *Saccharomyces*, *Trichosporon*, *Weissella*, *Candida*, *Torulaspora*, *Clavispora*, and *Wickerhamomyces* (Figure 4A). This indicates that flavor-producing microorganisms are not necessarily highly abundant. Compared to dominant species, these non-dominant microorganisms are more easily overlooked and harder to isolate. The flavor profiles generated by the core microbiota identified by taxonomy displayed compositional contours similar to, but not identical to, those of traditional fermentation [49]. Our findings may offer an explanation for this phenomenon, highlighting the critical contribution of flavor-producing yet non-dominant microorganisms to the formation of flavor compounds in fermented foods.

The application of SynComs represents an emerging trend in the development of traditional fermented foods, offering improved stability and safety in product quality [50]. Few studies have explored the development of SynCom for sesame flavor-type *baijiu* fermentation. Designing SynComs requires precise identification of core microbial members in complex fermentation. In this work, we propose a strategy for the construction of SynCom for sesame flavor-type *baijiu* fermentation (Figure 4A). The selection of SynCom members was based on the core microbiota identified through the integration of taxonomic composition, transcriptional activity, and functional genes associated with flavor compound diversity and sulfur compound production. The results from the simulated fermentation demonstrated that the constructed SynCom exhibited the best performance in flavor compound diversity and sulfur compound production compared to other groups (Figure 4D–F). With respect to sulfur compounds, our attention was focused on 3-(methylthio) propanal, which plays a vital role in sesame aroma and exhibits an ultra-low detection threshold of 7.12 μg/L [10]. Existing data on SynCom construction have primarily focused on replicating flavor profiles in situ, such as in *baijiu*, vegetable, and vinegar fermentations [19,20,21]. In contrast, the SynCom we constructed enhanced the flavor compound production of sesame flavor-type *baijiu*, including the diversity of flavor compounds and the content of sulfur compounds in large-scale production (Figure 5C,D). In addition, most studies of SynComs in traditional fermented foods remain at the preliminary design stage at the laboratory level [51,52]; our SynCom achieved the transition from laboratory-level experiments to large-scale production. Because many food fermentations share similar traits, the methods for constructing SynComs can also be used in a variety of food fermentations.

Our SynCom provides an opportunity to examine the mechanisms of species interaction. The top-performing SynCom (SC3) was composed of 13 microbial genera, with high-abundance species from each taxon selected for fermentation. Different species or strains within the same taxon can exhibit distinct metabolic functions [53,54]. Hence, future work should focus on collecting more functional strains. The initial inoculation ratio impacted microbial interactions and metabolic capacity [55,56]. In this work, we adopted the same inoculation ratio for the SynCom. Meanwhile, optimizing this ratio represents a critical avenue for enhancing fermentation performance in future research. Compared with the SC1 and SC2 SynComs, SC3 harbored more abundant bacterial and fungal genera. Our previous study demonstrated that fungal diversity promoted flavor compound formation, whereas bacterial diversity showed no such positive effect. Notably, bacterial–fungal interactions have been reported to exert positive effects on flavor compound formation [35]. For example, a synergistic effect on flavor complexity was discovered in the co-culture of no-*S. cerevisiae* and *L. plantarum* [57]. Accordingly, the superior performance of SC3 may therefore be attributed to its higher fungal diversity, together with the synergistic interactions between its more diverse bacteria and fungi. The SynCom in this work offers an opportunity to reveal the mechanism of bacterial and fungal interactions in the formation of flavor compounds.

## 5. Conclusions

In conclusion, this work identifies core microbiota through multi-omics analysis, defined by their taxonomic composition, transcriptional activity, and contributions to flavor compound diversity and sulfur production. Based on the core microbiota, we constructed a SynCom that not only enhanced the diversity of flavor compounds and the production of sulfur compounds but also maintained the stability of ethanol level observed in situ, thereby enabling its application in large-scale production. This work provides insights into designing SynComs to enhance the formation of flavor compounds in complex *baijiu* fermentations.

## Data Availability

Raw 16S rRNA amplicon sequencing data for bacteria and fungi were deposited in the DDBJ Sequence Read Archive under the accession numbers DRA011935 and DRA011936, respectively [22]. Raw metatranscriptomic data were also available in the DDBJ under accession number DRA008889 [23]. Additionally, raw metagenomic data were deposited in the NCBI Sequence Read Archive under the accession number PRJNA1208951 [13].

## Supplementary Materials

The following supporting information can be downloaded at: https://www.mdpi.com/article/10.3390/foods15091476/s1. Figure S1: Composition of flavor compounds from SC3 and the control group in large-scale production. The best-performing SynCom (SC3) was selected to be made into Fuqu for large-scale fermentation. Fermentations using commercial *Fuqu* were designated as the control group. The SC3 and control fermentations were carried out in biological triplicates. The data for flavor compounds were Z-score transformed; Table S1: Identification information of isolated strains.
