# Supplementary material for: Designing a Synthetic Microbial Community to Enhance Flavor Compound Production in Sesame Flavor-Type Baijiu Fermentation
Source: Foods. 2026 Apr 23;15(9):1476. doi: 10.3390/foods15091476 (PMC13163537; doi:10.3390/foods15091476)

Supplemental Information

Supplementary figures captions:

Figure. S1 Composition of flavor compounds from SC3 and the control group in large-scale production. The best-performing SynCom (SC3) was selected to be made into *Fuqu* for large-scale fermentation. Fermentations using commercial *Fuqu* were designated as the control group. The SC3 and control fermentations were carried out in biological triplicates. The data for flavor compounds were Z-score transformed.

Supplementary Figure S1

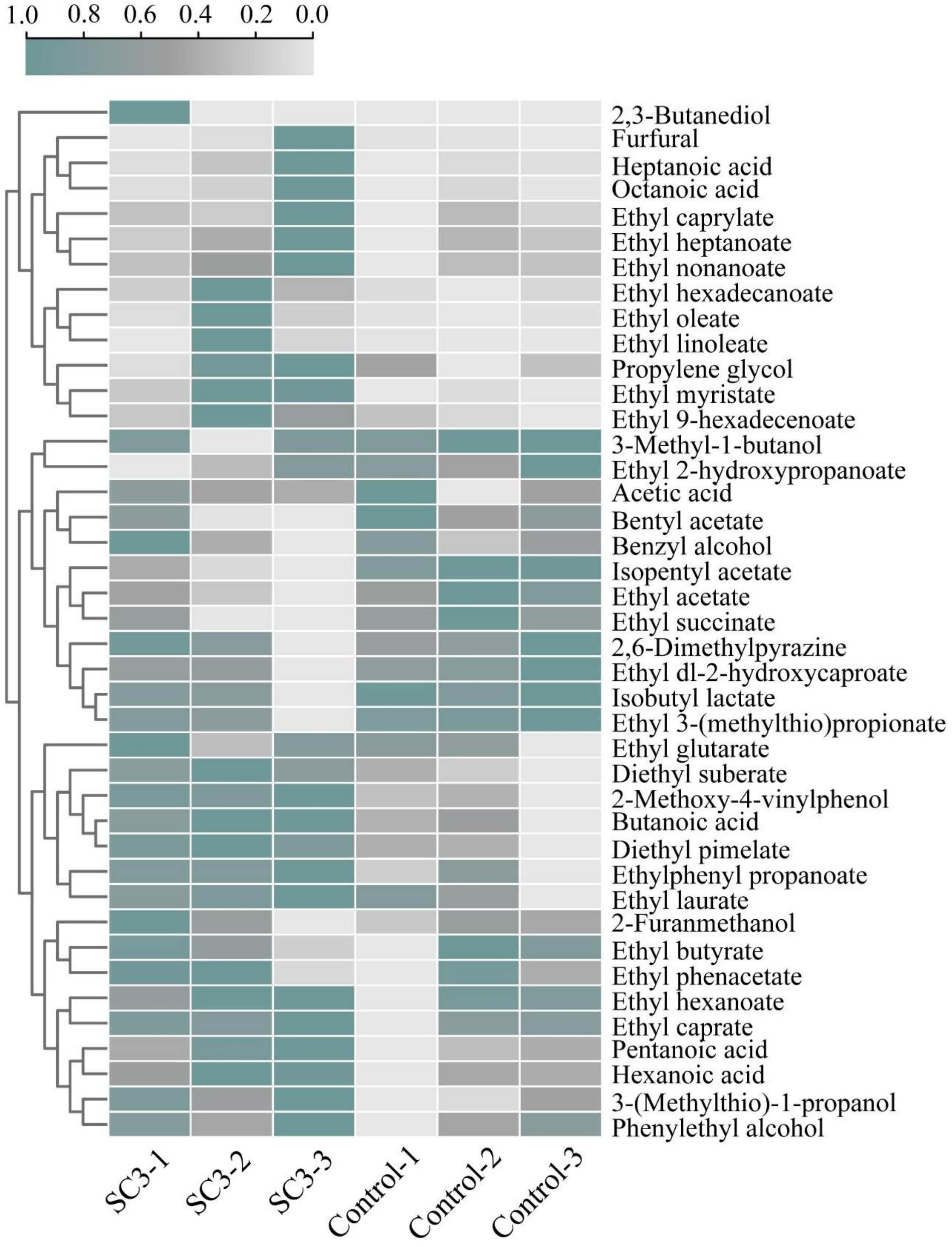

Supplement: Supplementary file 1 [file foods-15-01476-s001.zip › figure S1.pdf]
